# Stress State Analysis and Failure Mechanisms of Masonry Columns Reinforced with FRP under Concentric Compressive Load

**DOI:** 10.3390/polym8050176

**Published:** 2016-04-29

**Authors:** Jiří Witzany, Radek Zigler

**Affiliations:** Department of Building Structures, Czech Technical University in Prague, Faculty of Civil Engineering, Thákurova 7, 166 29 Prague, Czech Republic; witzany@fsv.cvut.cz

**Keywords:** masonry, brick, stone, CFRP, CFRCM, external reinforcement, near surface reinforcement, experimental testing

## Abstract

The strengthening and stabilization of damaged compressed masonry columns with composites based on fabrics of high-strength fibers and epoxy resin, or polymer-modified cement mixtures, belongs to novel, partially non-invasive and reversible progressive methods. The stabilizing and reinforcing effect of these fabrics significantly applies to masonry structures under concentric compressive loading whose failure mechanism is characterized by the appearance and development of vertical tensile cracks accompanied by an increase in horizontal masonry strain. During the appearance of micro and hairline cracks (10^−3^ to 10^−1^ mm), the effect of non-pre-stressed wrapping composite is very small. The favorable effect of passive wrapping is only intensively manifested after the appearance of cracks (10^−1^ mm and bigger) at higher loading levels. In the case of “optimum” reinforcement of a masonry column, the experimental research showed an increase in vertical displacements δ_y_ (up to 247%), horizontal displacements δ_x_ (up to 742%) and ultimate load-bearing capacity (up to 136%) compared to the values reached in unreinforced masonry columns. In the case of masonry structures in which no intensive “bed joint filler–masonry unit” interaction occurs, e.g., in regular coursed masonry with little differences in the mechanical characteristics of masonry units and the binder, the reinforcing effect of the fabric applies only partially.

## 1. Introduction

In the last two decades, fiber reinforced polymer (FRP) materials have found their place among traditional reinforcement and stabilization methods for masonry structures [1]. Strengthening with high-strength carbon fibers belongs to novel, partially non-invasive and reversible progressive methods based on limiting the appearance and development of tensile cracks resulting from horizontal tensile stresses caused by the contraction and mutual interaction of masonry components with different displacement properties. These are, above all, applications for strengthening structures against bending, tension, and shear and, to a lesser extent, in increasing the load-bearing capacity by wrapping the load-bearing members (mainly columns and pillars [2,3]). FRP materials also play an important role in strengthening structures in terms of seismic safety [4,5,6,7].

FRP materials offer numerous advantages for strengthening and stabilizing historic, mainly masonry structures, by virtue of their low weight, high effectiveness, and potential reversibility [8]. To date, the application of FRP composites in the restoration of historic and heritage buildings has been primarily focused on stabilizing vertical load-bearing and vaulted structures to resist the effects of horizontal loads due to induced and natural seismicity [9,10].

Research into the reinforcement of masonry structures with FRP materials has only in recent years been aimed at increasing the load-bearing capacity of vertical structures (columns and pillars) by wrapping [11,12]. The bulk of research addresses brick masonry [11,13], with only a few research studies taking interest in stone masonry as well [14,15,16,17,18].

The bond strength between the reinforcing composite layer and the strengthened masonry is the key parameter significantly influencing the resultant effectiveness of the strengthening of masonry structures with FRP materials [19,20]. This issue has been the subject of extensive experimental [21,22] and theoretical research [23,24] during the last few years. Some research studies have been focused on the effect of the binder (mortar) on the resultant bond strength between the FRP material and the masonry substrate [25,26,27], the design of new and suitable theoretical models describing the behavior of FRP materials in terms of their adhesion to the substrate [28], or on the experimental and theoretical investigation of using new, flexible binders for gluing externally bonded FRP reinforcement [29].

## 2. Numerical Analysis and Experimental Verification of Failure Mechanisms in Compressed Masonry Columns

**Figure 1:**
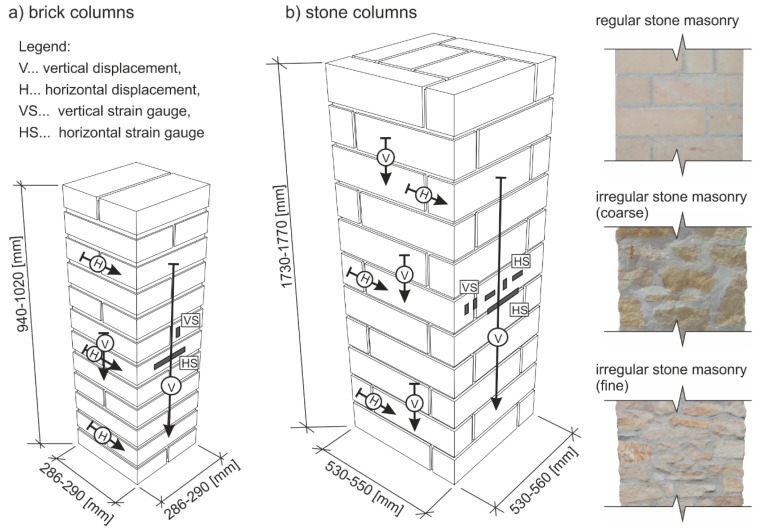
Scheme of test pieces (dimensions, measurement gauge positions. Arrows indicate the direction and length of measurement gauges). (**a**) Brick columns, (**b**) Stone columns.

**Figure 2:**
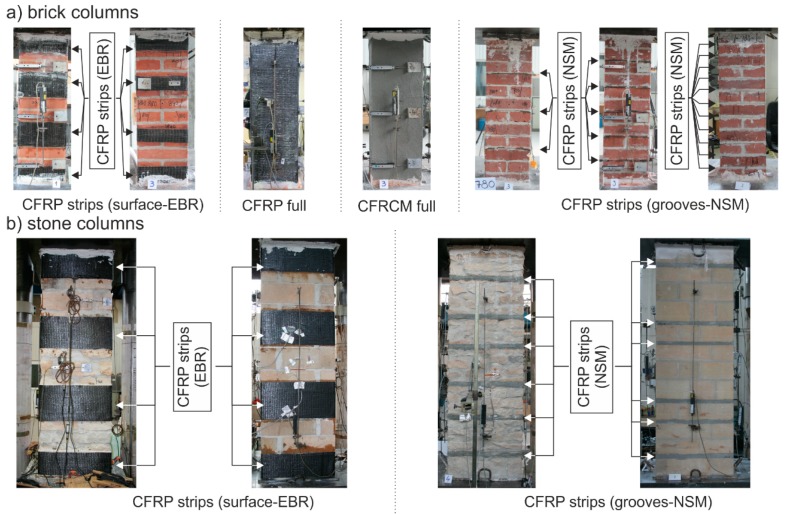
Test piece overview, (**a**) Brick columns; (**b**) Stone columns. CFRP, carbon fiber reinforced polymer; EBR, externally bonded reinforcement; CFRCM, carbon fiber reinforced cementitious matrix; NSM, near surface mounted reinforcement.

The experimental research was performed on test pieces of brick masonry with dimensions (width × thickness × height) of approximately 286–290 mm × 286–290 mm × 940–1020 mm and stone masonry with dimensions of approximately 530–550 mm × 530–560 mm × 1730–1770 mm with cement mortar as a binder (Figure 1). The test pieces included reference masonry columns without any FRP application, fabricated as “unreinforced”, *i.e.*, without passive wrapping of an FRP fabric-based composite, masonry columns “reinforced” with non-pre-stressed FRP-based composite strips applied to their surface with an adhesive bridge of either epoxy resin or a special polymer-modified cement mixture, or reinforced by overall wrapping, and masonry columns reinforced with FRP-based composite strips inserted into horizontal grooves cut into the masonry (Figure 2). An overview of test pieces used is presented in Table 1 and Table 2.

The main objective of the experimental research was to verify the effectiveness (strength performance) of different reinforcing methods according to their parameters (height and distance of reinforcing strips, the number and distance of reinforcing lamellae, influence of used materials or technology *etc.*). Special interest within the experimental research was focused on verifying the effectiveness of additional column reinforcement to tensile crack damage from the initial phase (*i.e.*, vertical tensile cracks passing through 3 to 6 rows of masonry). The reinforcing methods were chosen with the aim of minimizing possible interference into the structure (surface or near-surface application of FRP materials).

The columns were reinforced by wrapping them in non-pre-stressed strips of high-strength carbon fiber fabric (Tyfo^®^ SCH-41 fabric) glued onto the reinforced columns with Tyfo^®^ S thixotropic two-component epoxy resin (FRP system), or by non-pre-stressed strips of mesh made of high-strength carbon fiber Ruredil X Mesh C10 and inorganic stabilized cement matrix M25 (FRCM system). The material characteristics of these carbon fabrics, epoxy adhesive and cement matrix are presented in Table 3 and Table 4. The composite strips were 75 or 150 mm in height and were placed at 3 or 4 levels—at the top and bottom of the column and in the middle (3 levels, axial distance of strips was 450 mm) or at third distances along the column’s height (4 levels, axial distance of strips was 300 mm). Another case of reinforcement was overall wrapping of the column in a non- pre-stressed fabric. In this case, a continuous piece of fabric (Tyfo^®^ SCH-41 fabric) was placed over the whole height of the column (glued by Tyfo^®^ S epoxy resin). In the final case of reinforcement, on-site pre-laminated strips (lamellae) were inserted into grooves cut into the masonry (pre-laminated strip width was 40 mm, thickness was 5–7 mm and their surface was covered in fine quartz sand for better adhesion. Groove depth was 50–60 mm and thickness was 15–20 mm) filled with polymer cement mortar (Betosan Superfix TH f).

Prior to the application of reinforcing fabrics, the surface of the reinforced columns was levelled and cleaned, stripped of non-cohesive parts, and in the case of stonework columns of coursed rubble masonry and quarried sandstone (irregular sandstone blocks), the surface was levelled where the reinforcing fabric was to be applied. In all cases the column corners were rounded (with a fillet radius of 20 mm).

Reinforcement was performed using a fabric with a unidirectional arrangement of fibers applied in a single layer. Thus, the composite consisted of one layer of fabric and an outer and inner layer of epoxy resin. Both ends of the fabric were connected by an overlap (at least 250 mm in the case of clay brick masonry columns and 400 mm in the case of stone masonry columns). The shear strength of the epoxy resin was relevant for determining the anchoring length so that it would match the corresponding tensile strength of the composite strip. Experimental loading mostly resulted in the fabric’s failure across its surface area, not at the mutual connection of the ends of the fabric forming the overlap.

The test pieces were gradually loaded with incremental loading steps of 60 kN up to their failure. To verify the permanent displacement component, unloading to the basic loading value of 60 kN (10% of the assumed ultimate load of unreinforced columns of brick or stone masonry) was performed after each third loading step. The ultimate load on the columns was identified as a sudden drop in loading accompanied by a loss in the ability of the loaded member to transfer compressive loads as well as the destruction and disintegration of the column masonry. The testing device required the loading process to be controlled by force, thus the post peak phase of the column’s behavior could not be captured.

**Figure 3:**
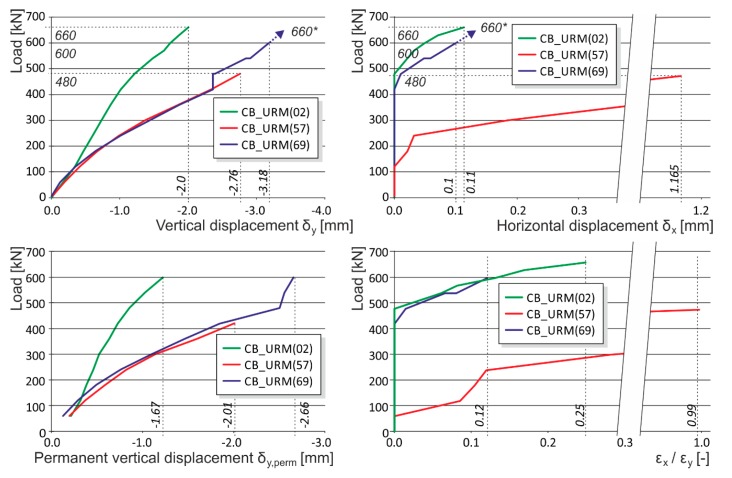
Experimentally obtained load displacement diagrams—unreinforced columns (CB_URM). * are the ultimate loads reached during the experimental testing. Arrows indicate that the corresponding displacement values are not measured due to the removal of measuring gauges before the last loading step (possible destruction).

**Figure 4:**
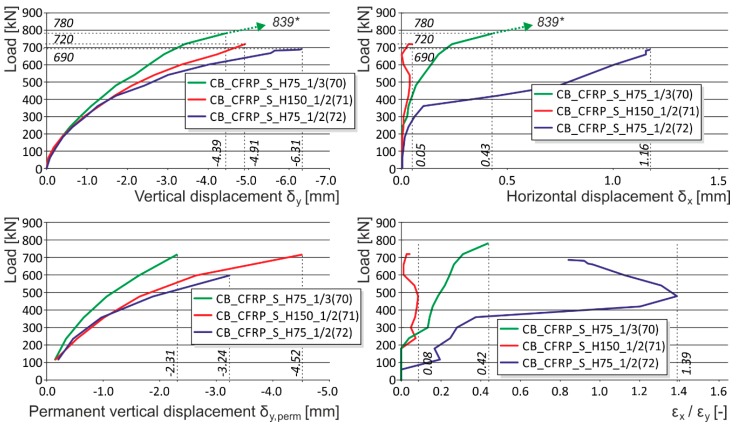
Experimentally obtained load displacement diagrams—strip placement and width influence (CB_CFRP_S_H75_1/3, CB_CFRP_S_H150_1/2, CB_CFRP_S_H75_1/2). * are the ultimate loads reached during the experimental testing. Arrows indicate that the corresponding displacement values are not measured due to the removal of measuring gauges before the last loading step (possible destruction).

**Figure 5:**
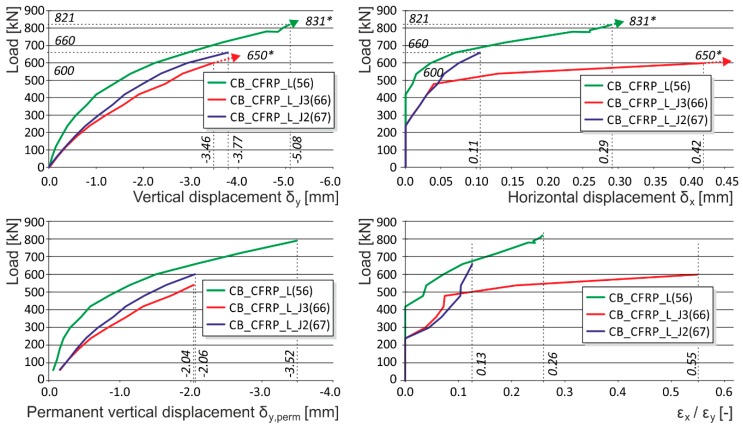
Experimentally obtained load displacement diagrams—NSM reinforcement placement (CB_CFRP_L, CB_CFRP_L_J3, CB_CFRP_L_J2). * are the ultimate loads reached during the experimental testing. Arrows indicate that the corresponding displacement values are not measured due to the removal of measuring gauges before the last loading step (possible destruction).

**Figure 6:**
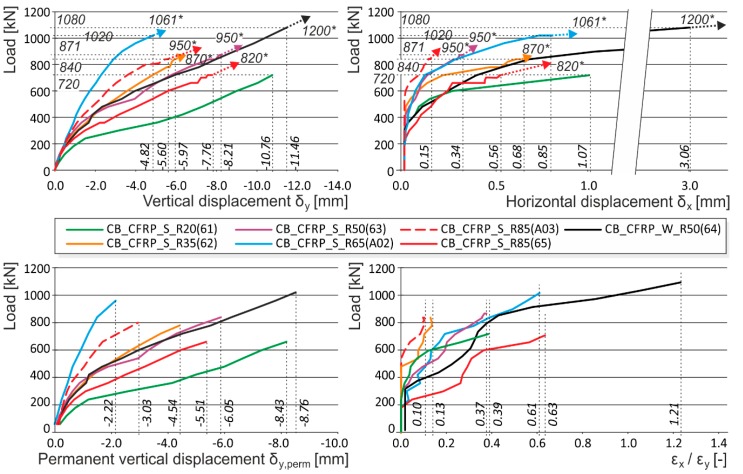
Experimentally obtained load displacement diagrams—corner radius influence (CB_CFRP_S_R20, CB_CFRP_S_R35, CB_CFRP_S_R50 CB_CFRP_S_R65, CB_CFRP_S_R85, CB_CFRP_W_R50). * are the ultimate loads reached during the experimental testing. Arrows indicate that the corresponding displacement values are not measured due to the removal of measuring gauges before the last loading step (possible destruction).

**Figure 7:**
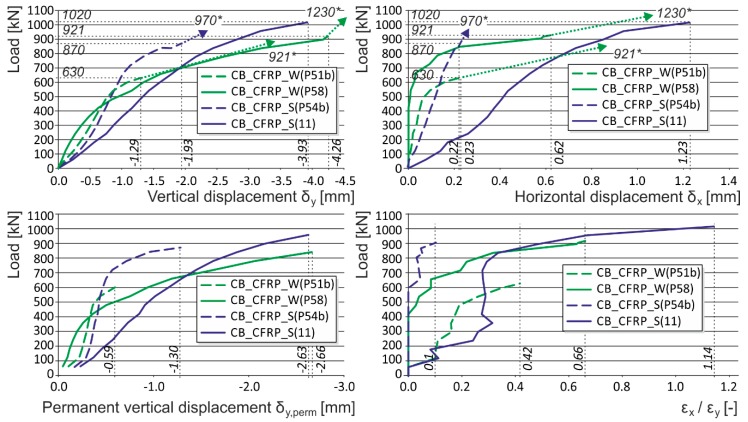
Experimentally obtained load displacement diagrams—initial crack influence (CB_CFRP_W, CB_CFRP_S). * are the ultimate loads reached during the experimental testing. Arrows indicate that the corresponding displacement values are not measured due to the removal of measuring gauges before the last loading step (possible destruction).

**Figure 8:**
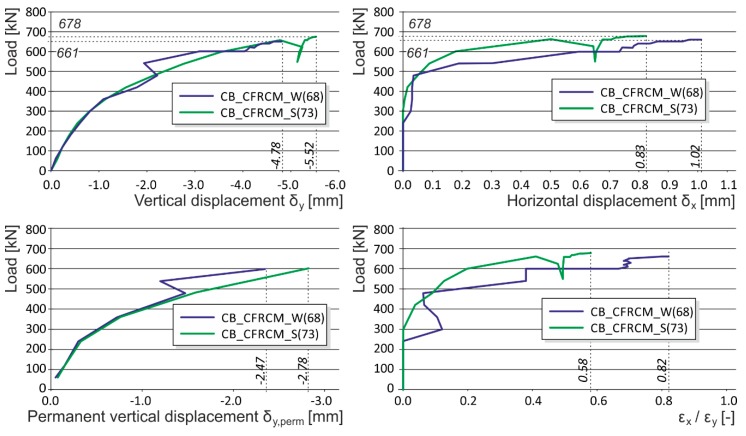
Experimentally obtained load displacement diagrams—FRCM reinforcement (CB_CFRCM_W, CB_CFRCM_S).

**Figure 9:**
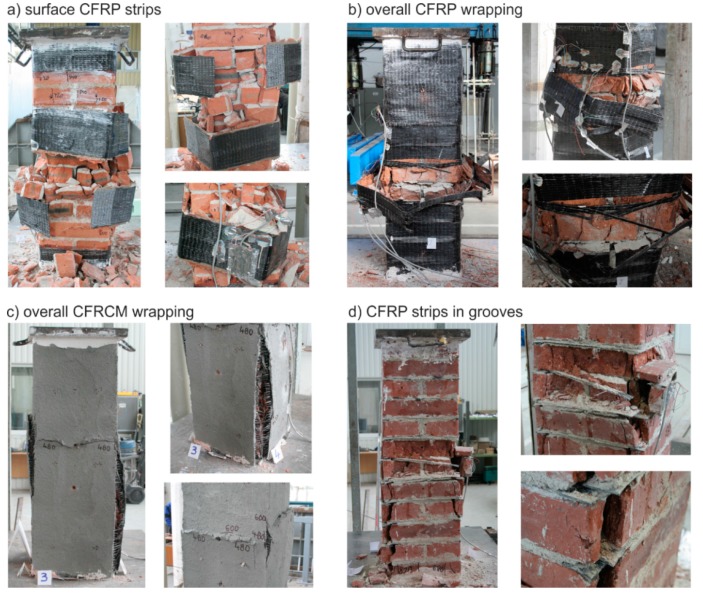
Characteristic failures of reinforced brick masonry columns at reaching ultimate load; (**a**) CFRP strips on surface (EBR); (**b**) overall CFRP wrapping; (**c**) overall CFRCM wrapping; (**d**) CFRP pre-laminated strips inserted into grooves (NSM).

**Figure 10:**
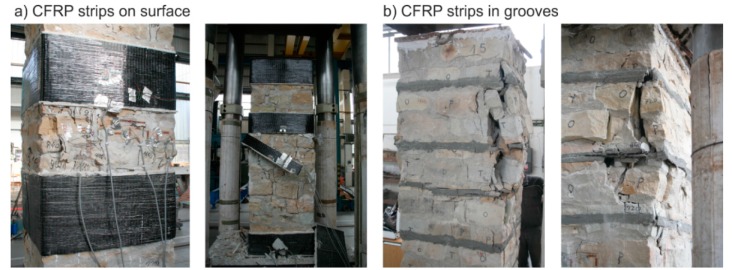
Characteristic failures of reinforced brick masonry columns at reaching ultimate load; (**a**) CFRP strips on surface (EBR); (**b**) CFRP pre-laminated strips inserted into grooves (NSM).

The masonry displacement and strain values during loading were monitored by means of linear displacement gauges (LVDT) and resistance strain gauges (see Figure 1). To avoid undesirable distortion of stress values obtained from the strain gauges, the minimum thickness of epoxy resin at the location of mounted strain gauges was strictly monitored. The displacement gauges were removed before reaching the ultimate load (in the last loading step) due to the possibility of their destruction. Partial results of the experimental research assessment are presented in Table 5 and Table 6, and graphically displayed in Figure 3, Figure 4, Figure 5, Figure 6, Figure 7 and Figure 8. Examples of characteristic masonry column failures at reaching the ultimate load are shown in Figure 9 and Figure 10.

The theoretical value of a column’s load bearing capacity was, in the case of unreinforced columns, identified pursuant to ČSN EN 1996 1-1 [30]:

(1) $$N_{\text{theor},\text{URM}}=\Phi_{i,m}\cdot f_{d}\cdot A_{m}\;[kN]$$

where Φ_i,m_ is the reduction factor at the top (bottom) or in the middle of the column reflecting slenderness end eccentricity [-], *f*_d_ is the design compressive strength of masonry [kPa], and *A*_m_ is the loaded horizontal gross cross-sectional area [m^2^].

(2) $$f_{d}=\frac{f_{k}}{r_{m}}\;[\text{kPa}]$$

where *f*_k_ is the characteristic compressive strength of masonry [kPa] according to the values for compressive strength of masonry units (*f*_b_) and mortar (*f*_m_), γ_m_ is the partial factor for masonry material [-], including mortar execution, moisture content, uncertainties in geometry, and overall degradation pursuant to ISO 13822:2010 [33].

At the top or bottom of the column

(3) $$\varphi_{i}=1-2\cdot \frac{e_{i}}{t}\;[-]$$

where *e*_i_ is the load eccentricity at the top or bottom [m], taking into account also the eccentricity (if any) resulting from horizontal loads (*e*_he_) and the initial eccentricity (*e*_init_) based on the effective height of the column (*h*_ef_/450), and *t* is the thickness of the column (dimension in the appropriate direction) [m].

In the middle of the column height

(4) $$\varphi_{m}=A_{1}\cdot e^{-\frac{u^{2}}{2}}\;[-]$$

where

(5) $$A_{1}=1-2\cdot \frac{e_{\text{mk}}}{t}\;[-]$$

(6) $$u=\frac{\lambda -0.063}{0.73-1.17\frac{e_{\text{mk}}}{t}}\;[-]$$

where

(7) $$\lambda =\frac{h_{\text{ef}}}{t_{\text{ef}}}\sqrt{\frac{f_{k}}{E}}\;[-]$$

(8) $$e_{\text{mk}}=e_{\text{hm}}+e_{\text{init}}+e_{k}\;[m]$$

where *e*_mk_ is the eccentricity in the middle height of the column [m], *e*_hm_ is the eccentricity at mid-height resulting from horizontal loads [m], *e*_init_ is the initial eccentricity (*h*_ef_/450) [m], and *e*_k_ is the eccentricity due to creep [m].

(9) $$e_{k}=0.002\cdot \varphi_{¥}\cdot \frac{h_{\text{ef}}}{t_{\text{ef}}}\sqrt{t\cdot e_{m}}[m]$$

In the case of FRP reinforced masonry columns, the theoretical value of load bearing capacity was identified pursuant to CNR-DT 200 R1/2013 [32]:

(10) $$N_{\text{theor},\text{CRFRP}}=\frac{1}{\gamma_{\text{Rd}}}\cdot A_{m}\cdot f_{\text{mcd}}\;[\text{kN}]$$

where γ_Rd_ is the partial factor [-] equal to 1.10 for a confined column, *A*_m_ is loaded horizontal gross cross-sectional area of confined member [m^2^], and *f*_mcd_ is the design compressive strength of the FRP confined member.

(11) $$f_{\text{mcd}}=f_{\text{md}}\left[1+k^{'}\cdot {\left(\frac{f_{1,\text{eff}}}{f_{\text{md}}}\right)}^{\alpha_{1}}\right]\;[{\text{kP}}_{a}]$$

where *k’* is the non-dimensional coefficient [-], *f*_md_ is the design compressive strength of unconfined masonry [kPa], *f*_1,eff_ is the effective confining pressure [kPa], and *α*_1_ is the coefficient [-] equal to 0.5.

(12) $$k'=\alpha_{2}\cdot {\left(\frac{g_{m}}{1000}\right)}^{\alpha_{3}}\;[-]$$

where *g*_m_ is the masonry mass-density [kg/m^3^], and *α*_2_ and *α*_3_ are coefficients equal to 1.

(13) $$f_{1,\text{eff}}=k_{H}\cdot k_{V}\cdot f_{1}\;[\text{kPa}]$$

where *k*_H_ is the coefficient of horizontal efficiency [-], *k*_V_ is the coefficient of vertical efficiency [-], and *f*_1_ is the lateral confining pressure [kPa].

(14) $$f_{1}=\frac{2\cdot t_{f}\cdot b_{f}\cdot E_{f}}{\max\{b,h\}\cdot p_{f}}\cdot \varepsilon_{\text{fd},\text{rid}}\;[\text{kPa}]$$

where *t*_f_ is the FRP thickness [m], *b*_f_ is the FRP strip width [m], *p*_f_ is the center-to-center spacing of FRP strips [m], *E*_f_ is the Young modulus of FRP sheet elasticity [kPa], ε_fd,rid_ is the reduced design value of FRP strain measured at a columns collapse [-], and *b* and *h* are the column’s cross-sectional dimensions [m].

(15) $$k_{H}=1-\frac{b^{'2}+h^{'2}}{3\cdot A_{m}}\;[-]$$

where *b’* and *h’* are the column’s dimensions reduced by the corner radius (*b* − 2*r*_c_).

(16) $$k_{V}={\left(1-\frac{p_{f}}{2\cdot \min\{b,h\}}\right)}^{2}\;[-]$$

for continuous confinement k_V_ is equal to 1

(17) $$\varepsilon_{\text{fd},\text{rid}}=\min\{\eta_{s}\cdot \varepsilon_{\text{fk}}/\gamma_{f};0.004\}\;[-]$$

where η_s_ is the environmental conversion factor [-], ranging between 0.85 to 0.95 for carbon fibers bonded with epoxy resin (internal or external exposure, respectively), *ε*_fk_ is the ultimate strain of FRP sheets [-], γ_f_ is the partial factor of FRP sheets [-] (ranging between 1 and 1.50, depending on the limit state and on the failure mode—whether debonding occurs or not), and 0.004 is the conventional strain limit.

Note: The following load displacement diagrams are presented without the unloading branches (made during the loading process after each third loading step) for better readability. The vertical displacement plotted is a mean value of two measurements from opposite sides of the columns (long measuring bases were used for capturing the overall masonry behavior). Horizontal displacement was measured at the middle height of the column, where the biggest horizontal displacement of the column was expected.

### 2.1. Failure Mechanism of Brick and Stone Masonry Columns under Concentric Compressive Load

**Figure 11:**
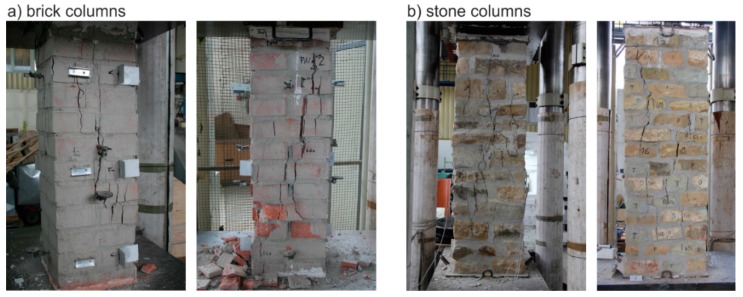
Typical failure of unreinforced compressed masonry columns; (**a**) brick columns; (**b**) stone columns.

**Figure 12:**
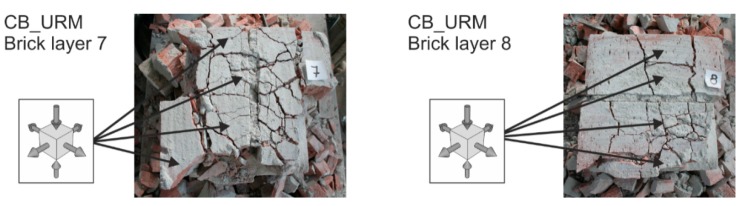
Typical failure of unreinforced compressed masonry columns—horizontal section.

Clarification of brickwork and stonework column failure mechanisms relative to their loading and layout is the basic starting-point for designing masonry structures, their reinforcement and method for stabilization, as well as for deriving accurate formulae for identifying their ultimate loads. Based on analysis of the experimental test results and numerical analyses, the following conclusions about the failure mechanism of compressed masonry columns under concentric load were drawn:

- The masonry failure process under concentric compressive load—its failure mechanism and a gradual loss in its ability to transfer loads—occurs in one or two phases with a different failure mechanism seen in each of these phases.
- In the phase when cracks appear and develop (phase I), failure due to “tension and shear” mostly applies (cracks arise in the direction of the principal compressive stress). In the second phase of progressive development and expansion of cracks (phase II), it is masonry failure due to “compression” that applies. In the case of unreinforced or weakly (“ineffectively”) reinforced concentrically compressed masonry structures with different masonry unit and binder properties, the failure mechanism solely or largely includes phase I. The appearance and development of vertical tensile and shear cracks precedes the ultimate load of masonry in compression being reached and is accompanied by the disruption of the integrity (rupture) of the column due to the effect of transverse tensile forces (Figure 11).
- During the initial phase I, when tensile and shear cracks running in the direction of compression trajectories arise and gradually develop throughout the masonry, redistribution and non-uniform distribution of compressive normal stresses in the horizontal cross sections of masonry appear. The transverse (horizontal) tensile (or shear) stresses cause gradual “subdivision” of the column into individual parts defined by vertically running tensile and shear cracks. During this process, the tensile strength of masonry units primarily applies (Figure 12).

### 2.2. Failure Mechanism of FRP Reinforced Columns

**Figure 13:**
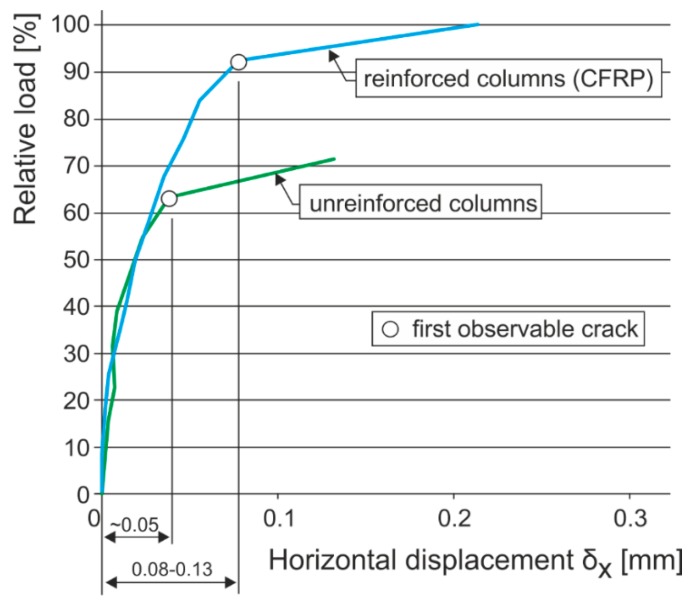
Horizontal displacement comparison of compressed reinforced and unreinforced structures.

Based on analyses of the experimental research results, we may say that in the initial failure phase—the appearance of micro and hairline cracks (10^−3^ to 10^−1^ mm in width)—in agreement with the “masonry–composite” interaction pattern and the relatively low tensile strength of ceramic or natural masonry units and other influences, the effect of a non-pre-stressed wrapping composite is very low (Figure 13). It is only after the appearance of cracks (in the order of 10^−1^ mm or bigger) under higher loading levels that the favorable effect of passive wrapping of column masonry applies more intensely.

**Figure 14:**
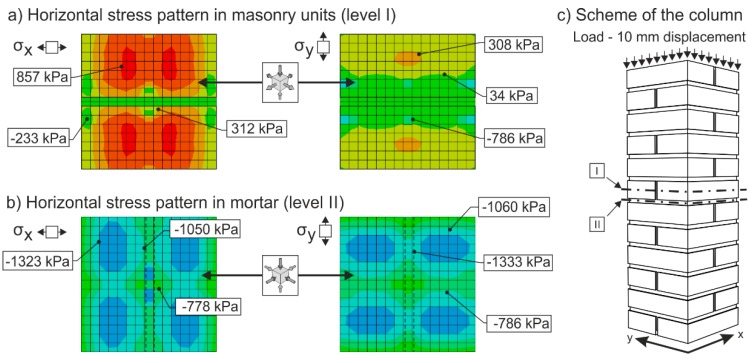
Stress pattern in an unreinforced masonry column. The colors in picture indicate the stress levels in the cross section of the column. Red, orange and yellow colors mean tensile stresses, green and blue colors mean compressive stresses. I and II (levels) are positions of the cross sections.

**Figure 15:**
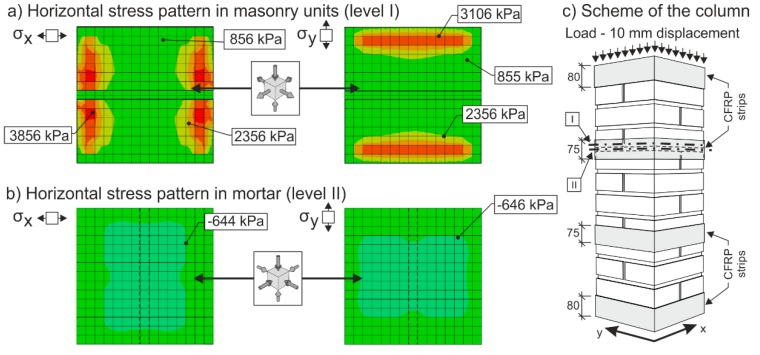
Stress pattern in a reinforced (CFRP strips, EBR) masonry column. The colors in picture indicate the stress levels in the cross section of the column. Red, orange and yellow mean tensile stresses, green and blue mean compressive stresses. I and II (levels) are positions of the cross sections.

- In the case of masonry columns weakly (“insufficiently”) reinforced with partial non-pre-stressed fabric-based composite wrapping strips, the gradual propagation and development process of tensile and shear cracks in the column masonry (phase I) is accompanied by a gradual growth in tensile stresses in this composite due to the effect of “expanding” masonry. Horizontal transverse displacement of masonry is caused by transverse stresses due to the effect of masonry contraction and the effect of mutual interaction between masonry units and the bed joint filler. Despite the growing horizontal and vertical displacements δ_x_ and δ_y_, the masonry column is still able to transfer the growing compressive load due to its reinforcement (stabilization) with discrete passive wrapping in a composite that takes up part of the tensile stresses (Figure 14 and Figure 15).

Figure 14 and Figure 15 represent the results of an Abaqus [34] linear FEM analysis. Masonry was modelled as two separate materials—bricks (*E*_bricks_ = 2500 MPa, η_bricks_ = 0.2) and mortar (*E*_mortar_ = 500 MPa, η_mortar_ = 0.15)—with solid three-dimensional hexahedral finite elements (8-node trilinear brick—C3D8R), and reinforcing FRP strips (*E*_mortar_ = 140000 MPa, η_mortar_ = 0.3) modelled with hexahedral shell elements (SC8R). The column was supported at the bottom by prescribed displacement and rotational constraints. The load was applied in the form of imposed vertical displacement at the top of the column.

**Figure 16:**
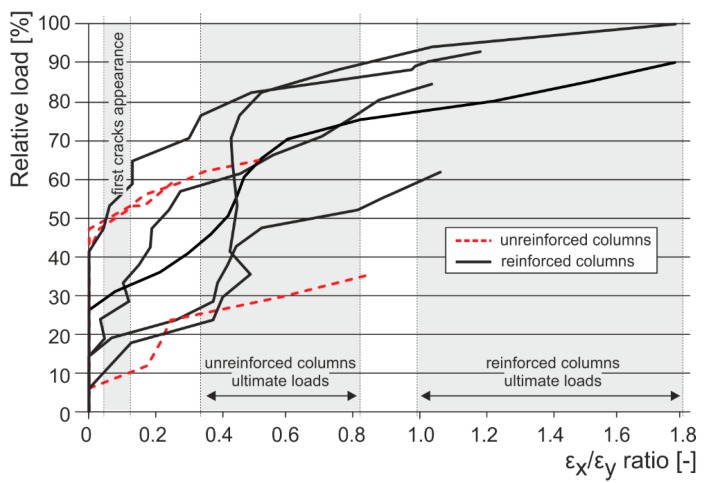
Vertical to horizontal strain ratio comparison of compressed reinforced and unreinforced structures—pseudo-ductility growth.

**Figure 17:**
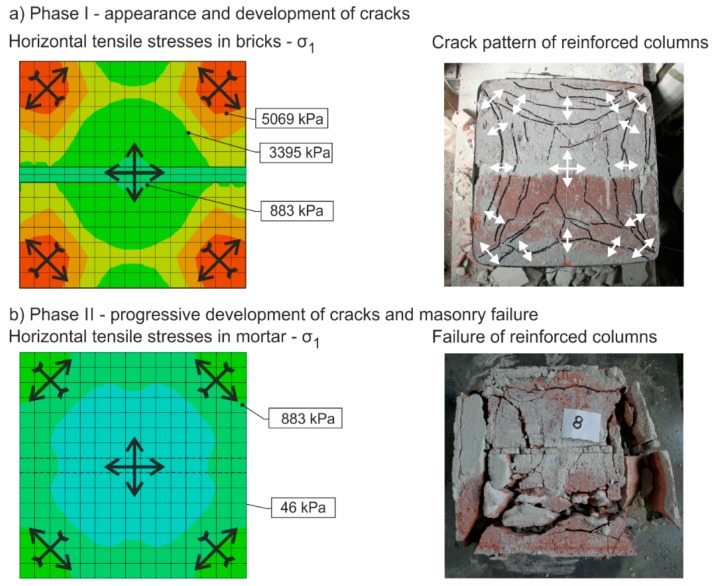
Characteristic failure mode of masonry units and the bed joint filler due to tensile cracks and crushing; (**a**) Phase I—appearance and development of cracks; (**b**) Phase II—progressive development and masonry column collapse (for Abaqus [34] numerical analysis detail see Figure 14). The colors in picture indicate the stress levels in the cross section of the column. Red, orange and yellow colors mean tensile stresses, green and blue colors mean compressive stresses. Arrows indicate the direction of the stresses (tensile and compressive).

- After tensile crack appearance (in the order of 10^−1^ mm and bigger), there is progressive growth in horizontal strains ε_x_, the propagation and development of tensile cracks, which become the major agents in the failure of masonry structures under compressive load. The initial value of the ε_x_/ε_y_ ratio in unreinforced columns in the phase preceding the appearance of the first (hairline) cracks lies in the interval of 0.05 to 0.1. When ultimate load in concentric compression is reached, the value of the ε_x_/ε_y_ ratio in unreinforced columns lies in the interval of 0.35 to 0.8, while in the case of columns reinforced with carbon fiber composites lies in the interval of 1.0 to 1.8 (Figure 16).
- In the case of concentrically compressed masonry columns effectively reinforced (strengthened) with a composite based on high-strength fibers and epoxy resin (which takes up part of the transverse tensile forces in the masonry due to the effect of the forced masonry displacement), the failure mode corresponding to phase II applies during the stage when the ultimate load in compression is approached and reached. In this phase of column masonry failure, the compressive strength of the masonry units applies to a greater extent. Attainment of ultimate load is accompanied by the disintegration and crushing of the masonry units and the bed joint filler and, successively, by the total failure (disintegration) of the masonry.
- Masonry failure due to the exhaustion of masonry unit compressive strength was observed, for example, in coursed masonry stonework columns with irregular masonry units and thin bed joints (*ca.* 12–15 mm), and in brickwork or stonework columns with effective wrapping in carbon fabric composites during the experimental research. Figure 17 displays the characteristic failure mode of masonry units and the bed joint filler due to tensile cracks and crushing. It is the consequence of efficient interaction between the carbon fabric wrapping and masonry during the phase of active vertical tensile crack development in the column masonry and the partial failure and disintegration of masonry units (phase I and phase II).
- In phase II, the adhesive bond in the contact joint between the carbon fabric and masonry usually fails, with the carbon fabric’s reaction to the column masonry’s forced displacement exerted by growing horizontal displacements concentrated in the areas close to the column’s edges. First, perimeter parts of the column loaded with biaxial stress states are damaged—separated by tensile cracks. In the central part of the masonry cross section exposed to stress states by triaxial compression/tension, the masonry completely disintegrates (mainly the masonry units). In this failure mechanism, the compressive strength of individual components (mainly masonry units) primarily applies. This failure mechanism mainly applies in cases with effective wrapping of a masonry column—overall wrapping or wrapping in strips optimally distributed along the column’s length. With increasing distance between and lower height of carbon fabric wrapping strips, as well as lower efficiency of wrapping strips in which lower ultimate load values in concentric compression are reached, the failure mechanism occurring during the first phase (column failure due to vertical tensile cracks) predominantly applies.

### 2.3. Passive FRP Wrapping Efficiency

**Figure 18:**
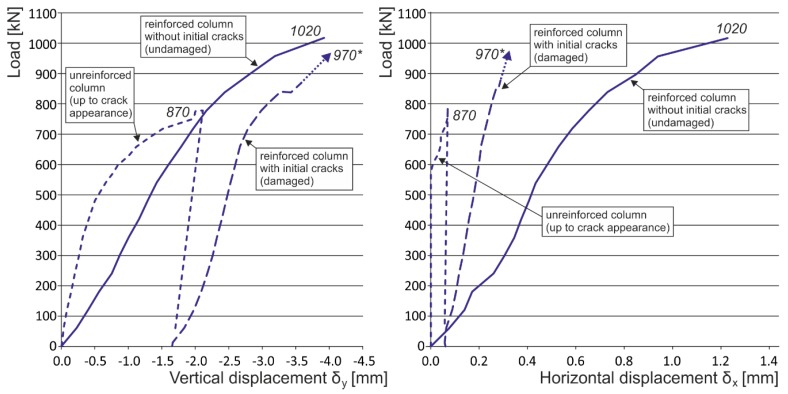
Experimentally obtained load displacement diagrams—masonry columns reinforced with CFRP strips (EBR) with and without the initial cracks. * are the ultimate loads reached during the experimental testing. Arrows indicate that the corresponding displacement values are not measured due to the removal of measuring gauges before the last loading step (possible destruction).

**Figure 19:**
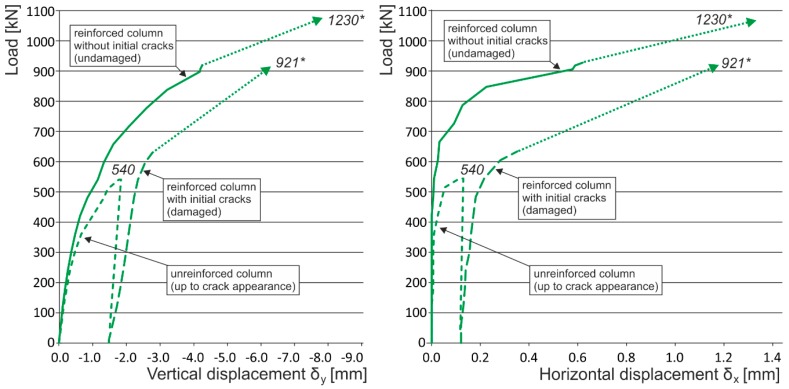
Experimentally obtained load displacement diagrams—masonry columns reinforced with overall CFRP wrapping with and without the initial cracks. * are the ultimate loads reached during the experimental testing. Arrows indicate that the corresponding displacement values are not measured due to the removal of measuring gauges before the last loading step (possible destruction).

**Figure 20:**
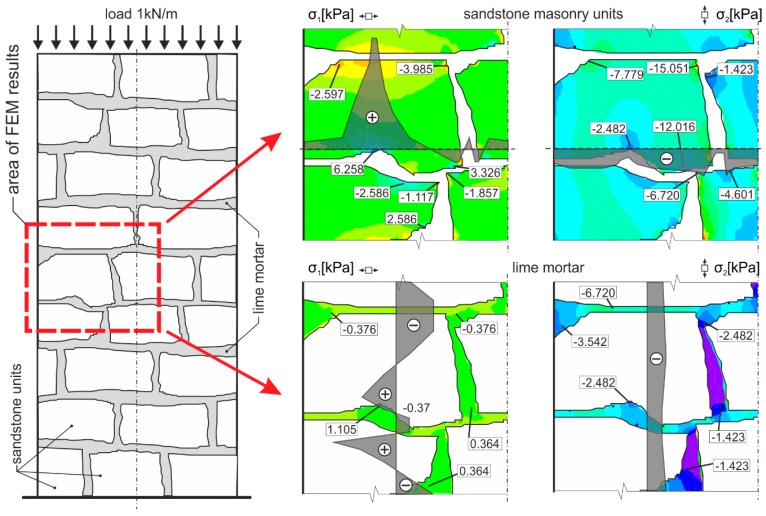
Critical points of vertical and horizontal concentration of stresses in masonry made of irregular masonry units (sandstone blocks and lime mortar; linear finite element (FEM) analysis using FEAT2000 [35] software. Masonry was modelled as two materials—stone blocks (*E*_sandstone_ = 15000 MPa, η_sandstone_ = 0.2) and mortar (*E*_mortar_ = 1500 MPa, η_mortar_ = 0.15)—with plane triangular elements. The column was supported at the bottom by prescribed displacement and rotational constraints. Load was applied as linear vertical force at the top of the column). The colors in picture indicate the stress levels in the column. For the σ_1_ principal stress in horizontal direction, orange, yellow and light green colors mean compressive stresses, dark green color means tensile stresses, for the σ_2_ principal stress in vertical direction, orange, yellow and light green colors mean higher values of compressive stresses and light blue, blue, dark blue and purple mean lower values of compressive stresses.

**Figure 21:**
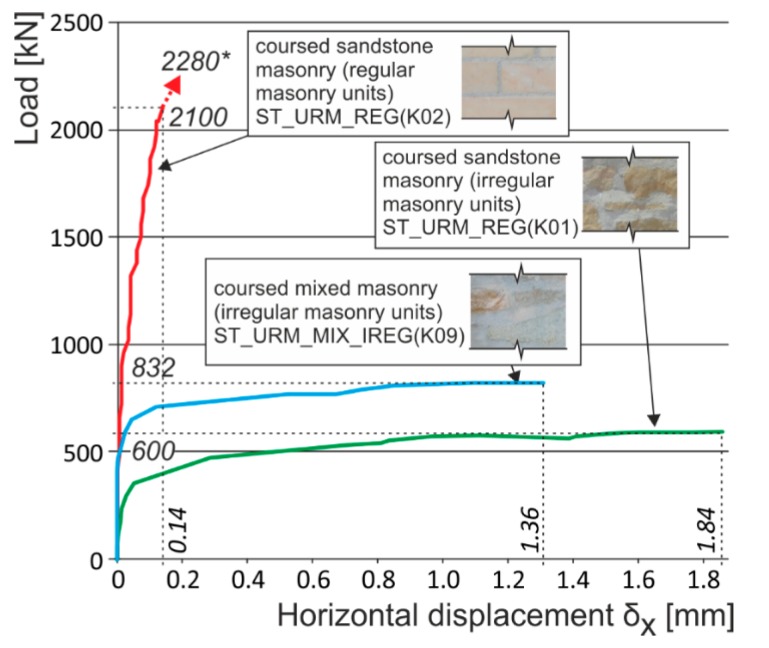
Experimentally obtained load displacement diagrams for different types of masonry units (shape and material).

**Figure 22:**
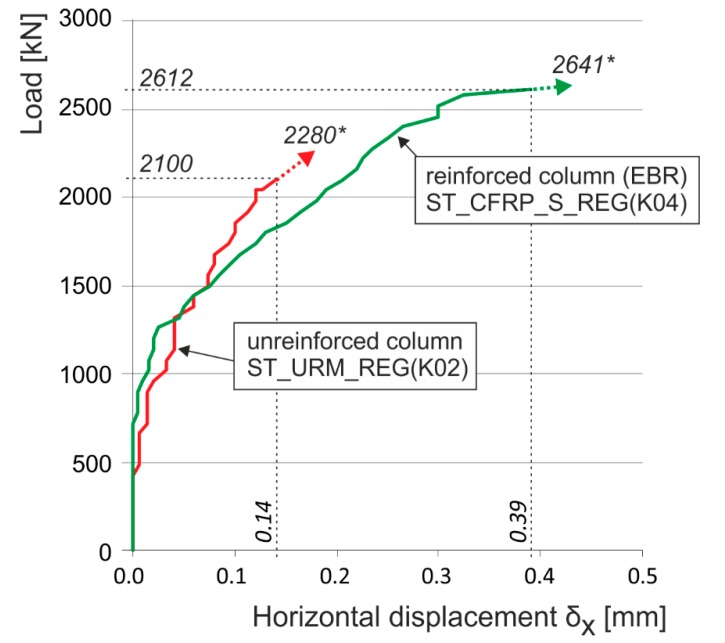
Experimentally obtained load displacement diagrams of unreinforced and reinforced (CFRP, EBR) stone masonry columns—regular masonry units. * are the ultimate loads reached during the experimental testing. Arrows indicate that the corresponding displacement values are not measured due to the removal of measuring gauges before the last loading step (possible destruction).

The experimental research into brick masonry columns pointed out the option of enhancing the displacement and strain characteristics and the ultimate load (see Table 5) of masonry columns under concentric compressive load reinforced with a composite of passive wrapping based on high-strength fabrics:

- In the case of “optimum” reinforcement of a masonry column with composite strips, *i.e.*, with a distance between strips smaller than or equal to no more than 1.5 times the composite strip height, there was an increase in vertical displacements δ_y_ to 247%, horizontal displacements δ_x_ to 742%, and ultimate load to 136% compared to the values reached for unreinforced masonry columns.
- In the case of overall wrapping of a masonry column in carbon composites, where the failure mechanism (corresponding to phase II) prior to reaching the ultimate load may be assumed, the growth in vertical displacements δ_y_ amounted to 161%, horizontal displacements δ_x_ 400%, and ultimate load 179%, compared to the values for unreinforced masonry columns.
- The experimental research into masonry columns damaged (before reinforcement) by tensile cracks up to (<1.5 mm) in width and extending to *ca.* 30%–40% of the column’s height and then additionally reinforced with FRP composites, pointed out the possibility of achieving the comparable mechanical and strain characteristics of identically reinforced undamaged masonry columns (without the initial cracks) through this additional reinforcement (Figure 18). This is particularly evident in phase I of the columns failure. In this phase, the transverse wrapping of a masonry column in composites with high load-bearing capacity is relevant and the effect of overall column reinforcement is especially dramatic (Figure 19). This finding agrees with the “composite–masonry” interaction pattern during which the composite is activated with effect only during the phase when tensile cracks develop (10^−1^ mm and bigger).
- The passive wrapping of column masonry damaged by tensile and shear cracks in non-pre-stressed fabrics made from high-strength fibers represents an efficient stabilization means by preventing further propagation and development of cracks, and securing the structural function of masonry damaged by tensile cracks.
- The effectiveness of column masonry passive wrapping in a carbon fiber composite depends on the extent of damage to individual columns and must be assessed separately for each case of masonry failure.
- The predominantly positive effect on stress states in triaxial compression of mortar in bed joints in brick masonry after passive wrapping cannot be applied to irregular stone or mixed masonry comprising irregular, roughly dressed masonry blocks with fragments and sharp-edged, undressed, quarry stone masonry units. Here, cracking and masonry damage occur in areas where local stress states with extreme stress values (σ_x_, σ_y_, τ_xy_) arise, e.g., around the masonry layout of irregular, sharp-edged units with a relatively higher modulus of elasticity than the surrounding relatively more yielding binder (Figure 20). These effects are usually unfavorably manifested, mainly at higher masonry loading values in compression (under loads exceeding 60% of ultimate load). As a consequence, these effects reduce the ultimate strength of stone masonry in compression (Figure 21).
- A special category is represented by compressed masonry columns in which, due to the properties and shape of masonry units, their binder and layout (regular masonry units with identical mechanical properties, thin bed joints with a filler of approximately the same mechanical characteristics as those of the masonry units), no transverse tensile forces that usually compensate the tendency of individual masonry layers towards different transverse displacements under concentric compressive load arise. In these reinforced masonry columns, the transverse displacement of masonry loaded by compressive forces is caused solely by contraction. The effectiveness of passive wrapping of column masonry in these cases is significantly lower compared to coursed masonry with quarry stone blocks (Figure 22).

### 2.4. Ultimate Strength of Masonry Reinforced with Composites under Concentric Compressive Load

**Figure 23:**
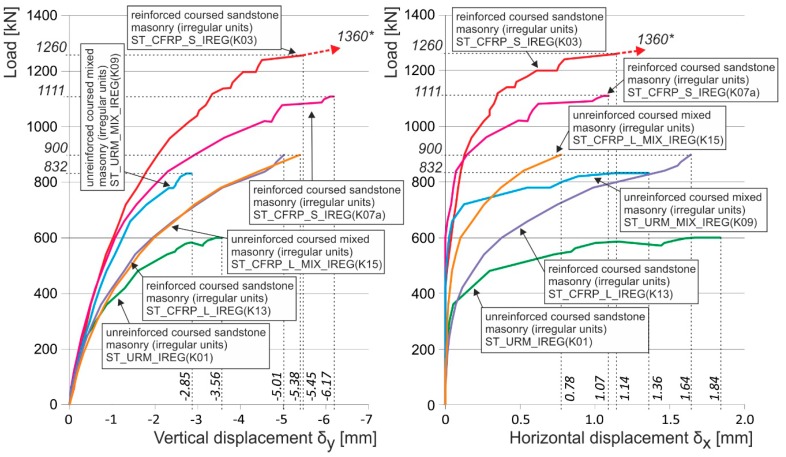
Experimentally obtained load displacement diagrams of unreinforced and reinforced (CFRP, EBR and NSM) stone masonry columns made from irregular masonry blocks. * are the ultimate loads reached during the experimental testing. Arrows indicate that the corresponding displacement values are not measured due to the removal of measuring gauges before the last loading step (possible destruction).

The analysis of the experimental research results and the failure mechanism of concentrically compressed masonry units indicated that the design procedure of masonry units practiced to-date based solely on the strength of individual masonry components in compression does not correspond to or encompass the interaction between individual masonry components and the masonry failure mechanism under concentric compressive load. The main drawback to calculation formulae may be the fact that standards (ČSN EN 1996-1-1 [30]) completely ignore or insufficiently consider the tensile strength of masonry components, which is the major agent in the appearance and development of tensile cracks. The full exploitation of tensile strength, masonry, or its individual components may be assumed in cases of coursed masonry with regular masonry units and a bed of low (≤15 mm) joint filler with approximately the same mechanical and displacement properties, and a good bond:

- In the case of columns of regular, coursed rubble masonry with dressed stone and thin bed joints reinforced by composite strips, the experimental research demonstrated that the tensile “capacity” of the composite was not efficiently exploited and, due to this, their ultimate load does not significantly increase. In the case of the above masonry, transverse displacements and forced transverse stresses due to the effect of passive wrapping are relatively very low as a consequence of the “mortar bed–masonry unit” lower intensity interaction.
- The ultimate load of a reinforced stonework column under concentric compressive load hardly differs from the ultimate load of an unreinforced column (see Figure 22). The failure occurs by reaching the ultimate displacement and strain of stone masonry in compression δ_ym_ accompanied by the local failure of masonry in compression—crushing and disintegration of masonry units and the binder. The experimentally measured, relatively low horizontal displacements δ_xm_ also agree with the failure mechanism of stonework columns with regular, dressed blocks and coursed masonry (phase II). This fact must be considered when calculating the compressive strength of stone masonry reinforced with FRP fabrics, in which the above described failure mechanism under compressive loading applies. Also, the relatively slight effect achieved by wrapping a column in fabrics of high-strength carbon or glass fibers may be objectively assumed.
- In the case of coursed or irregular stone masonry (column) with undressed or roughly dressed stone blocks and larger bed joints (>20–25 mm) under concentric compressive load, the application of the failure mechanism characterized by the appearance and development of tensile cracks running approximately in the direction of compression trajectories—phase I—may be assumed. It is usually accompanied by the gradual growth in transverse horizontal displacements δ_x_ in stone column masonry, together with a significant redistribution of compressive loading over the masonry cross section. In the case of an efficient “masonry–fabric (composite)” interaction, the ultimate load of stone masonry in compression dramatically rises compared to the ultimate load of unreinforced stone masonry (Figure 23) due to the effect of reinforcement by fabrics applied as wrapping bands (EBR) to the masonry or as strips inserted into horizontal joints (NSM).

## 3. Conclusions

The failure mechanism of compressed masonry structures is dramatically affected by the size and shape of masonry units, their mechanical characteristics and, last but not least, the masonry bond. For this reason, when calculating a reinforced masonry column’s ultimate load, specific cases of brick masonry structures, but mainly stone and historic structures, must be considered separately and the masonry layout (size of masonry units, bond, bed joint thickness, *etc.*) taken into account. Apart from accurately determining the mechanical properties of individual masonry components, a reliable specification of the theoretical value of masonry ultimate load requires respect towards the masonry failure mechanism. The stabilizing and reinforcing effect of fabrics based on inorganic fibers significantly applies to masonry structures (columns, walls) under concentric compressive load whose failure mechanism is characterized by the appearance and development of vertical tensile cracks accompanied by a growth in horizontal displacements of masonry. This effect is particularly pronounced in masonry where the masonry unit–mortar interaction applies (clay brick masonry with lime mortar) and in masonry with a stronger tendency to transverse deformation (irregular stone masonry with thicker mortar joints). To a lesser extent, this effect can be expected in masonry with a thin or zero bed joint or in masonry where mortar has the same characteristics as masonry units (cement mortar, *etc.*).

## Figures and Tables

**Table 1:** List of test pieces (clay brick columns), dimensions, and component strength.

Label	Dimensions [mm]	*f*_b_^1^ [MPa]	*f*_m_^2^ [MPa]	*f*_k_^3^ [MPa]	Reinforcement
CB_URM(02)	290 × 290 × 1020	15.0 ^4^	2.0 ^4^	4.51	Unreinforced (URM)
CB_CFRP_S(11)					CFRP strips on surface (EBR)
CB_URM(51a)					Unreinforced (URM), loaded up to first crack
CB_CFRP_W(51b)					CFRP overall wrapping with initial cracks
CB_URM(54a)	290 × 290 × 920				Unreinforced (URM), loaded up to first crack
CB_CFRP_S(54b)					CFRP strips on surface (EBR) with cracks
CB_CFRP_L(56)					CFRP strips in grooves (NSM), every bed joint
CB_URM(57)		17.28	1.5 ^4^	4.57	Unreinforced (URM)
CB_CFRP_W(58)		17.03	1.96	4.9	CFRP overall wrapping
CB_CFRP_S_R20(61)	286 × 286 × 870	11.60	1.88	3.70	CFRP strips on surface (EBR), column corner radius 20 mm
CB_CFRP_S_R35(62)			1.79	3.64	CFRP strips on surface (EBR), column corner radius 35 mm
CB_CFRP_S_R50(63)			1.84	3.67	CFRP strips on surface (EBR), column corner radius 50 mm
CB_CFRP_W_R50(64)			1.84	3.67	CFRP overall wrapping, column corner radius 50 mm
CB_CFRP_S_R85(65)			1.59	3.51	CFRP strips on surface (EBR), column corner radius 85 mm
CB_CFRP_L_J3(66)	290 × 290 × 890	11.60	1.23	3.25	CFRP strips in grooves (NSM), every 3rd bed joint
CB_CFRP_L_J2(67)				3.25	CFRP strips in grooves (NSM), every 2nd bed joint
CB_CFRCM_W(68)			1.5 ^4^	3.45	CFRCM overall wrapping
CB_URM(69)			2.24	3.89	Unreinforced (URM)
CB_CFRP_S_H75_1/3(70)	288 × 288 × 875		1.75	3.62	CFRP strips on surface (EBR), 75 mm at 1/3
CB_CFRP_S_H150_1/2(71)			1.68	3.57	CFRP strips on surface (EBR), 150 mm at 1/2
CB_CFRP_S_H75_1/2(72)			1.53	3.47	CFRP strips on surface (EBR), 75 mm at 1/2
CB_CFRCM_S(73)			1.5 ^4^	3.46	CFRCM strips on surface (EBR)
CB_CFRP_S_R65(A02)	286 × 286 × 890		2.08	3.81	CFRP strips on surface (EBR), column corner radius 65 mm
CB_CFRP_S_R85(A03)				3.81	CFRP strips on surface (EBR), column corner radius 85 mm

^1^ Experimentally obtained compressive strength of masonry units pursuant to ČSN EN 1996-1-1 [30]; ^2^ Experimentally obtained compressive strength of mortar pursuant to ČSN EN 1996-1-1 [30]; ^3^ Characteristic compressive strength of masonry pursuant to ČSN EN 1996-1-1 [30]; ^4^ Compressive strength value according to the manufacturer’s specification. Label explanation: CB—clay brick columns, URM—unreinforced, CFRP—reinforced with carbon fabric bonded by epoxy resin, CFRCM—reinforced with carbon mesh bonded by stabilized cement matrix, S—reinforcing strips, W—reinforced by overall wrapping, L—reinforced by lamellae inserted in horizontal grooves (bed joints), J—every Xth bed joint reinforced, H—height of reinforcing strip (mm), (X)—original number (in parenthesis) of the test specimen.

**Table 2:** List of test pieces (stone columns), dimensions, and component strength.

Label	Dimensions [mm]	*f*_b_^1^ [MPa]	*f*_m_^2^ [MPa]	*f*_k_^3^ [MPa]	Reinforcement
ST_URM_IREG(K01)	530 × 530 × 1750	22.73	2.0 ^4^	1.99 ^5^	Unreinforced (URM), coursed sandstone masonry (irregular units)
ST_URM_REG(K02)	545 × 545 × 1770	19.93		4.20	Unreinforced (URM), coursed sandstone masonry (regular units)
ST_CFRP_S_IREG(K03)	550 × 560 × 1735	22.73		1.99 ^5^	CFRP strips on surface (EBR), coursed sandstone masonry (irregular units)
ST_CFRP_S_REG(K04)	548 × 545 × 1760	19.93		4.20	CFRP strips on surface (EBR), coursed sandstone masonry (regular units)
ST_CFRP_S_IREG(K07a)	550 × 560 × 1735	19.51	3.03	0.94 ^5^	CFRP strips on surface (EBR), coursed sandstone masonry (irregular units)
ST_URM_MIX_IREG(K09)	550 × 560 × 1735	20.82	2.14	1.92 ^5^	Unreinforced (URM), coursed mixed (sandstone, marlstone) masonry (irregular units)
ST_CFRP_L_IREG(K13)	550 × 560 × 1730	19.51	2.25	0.86 ^5^	CFRP strips in grooves (NSM), coursed sandstone masonry (irregular units)
ST_CFRP_L_MIX_IREG(K15)	550 × 550 × 1730	22.84	1.29	1.93 ^5^	CFRP strips in grooves (NSM), coursed sandstone masonry (irregular units)

^1^ Experimentally obtained compressive strength of masonry units pursuant to ČSN EN 1996-1-1 [30]; ^2^ Experimentally obtained compressive strength of mortar pursuant to ČSN EN 1996-1-1 [30]; ^3^ Characteristic compressive strength of masonry pursuant to ČSN EN 1996-1-1 [30]; ^4^ Compressive strength value according to the manufacturer’s specification; ^5^ Characteristic compressive strength of masonry pursuant to ČSN 731101 (change B) [31]. Label explanation: ST—stone columns, URM—unreinforced, CFRP—reinforced with carbon fabric bonded by epoxy resin, S—reinforcing strips, L—reinforced by lamellae inserted in horizontal grooves, MIX—mixed stone masonry, REG—regular masonry units, IREG—irregular masonry units, (X)—original number (in parenthesis) of the test specimen.

**Table 3:** Material characteristics of carbon fabric and epoxy resin (FRP).

Material characteristic	Dry fibers	Epoxy resin	Composite properties
Ultimate Tensile Strength in Primary Fiber Direction (MPa)	3790	72.4	986
Tensile Modulus (GPa)	230	3.18	95.8
Elongation at Break (%)	1.7	5.0	1.0
Ultimate Tensile Strength Normalized by Thickness (MPa/mm)	–	–	986
Tensile Modulus Normalized by Thickness (GPa/mm)	–	–	95.8
Nominal Laminate Thickness (mm)	–	–	1.0
Flexural Strength (MPa)	–	123.4	123.4
Flexural Modulus (GPa)	–	3.12	3.12

Note: All values according to the manufacturer.

**Table 4:** Material characteristics of carbon mesh and cement matrix (FRCM).

Material characteristic	Dry fibers	Cement matrix
Ultimate Tensile Strength in Primary Fiber Direction (MPa)	4800	8.6 ^1^
Ultimate Compressive Strength (MPa)	–	25.56 ^1^
Tensile Modulus (GPa)	240	–
Elongation at Break (%)	1.8	–
Density (g/cm^3^)	1.78	–
Thickness (mm)	0.047	–

All values according to the manufacturer; ^1^ Experimentaly obtained values pursuant to ČSN EN 1996-1-1 [30].

**Table 5:** Summary of experimental loading results—clay brick columns.

Label	Ultimate vertical displacement ^1^ [mm]	Ultimate horizontal displacement ^1^ [mm]	*N*_theor_^4^ [kN]	*N*_exp_ [kN]	*N*_exp_/*N*_theor_
CB_URM(02)	2.0	0.11	341.4	660	1.93
CB_CFRP_S(11)	3.93	1.23	634.6	1,020	1.61
CB_URM(51a*)* ^2^	– ^2^	– ^2^	– ^3^	540 ^2^	– ^3^
CB_CFRP_W(51b)	1.29	0.22	727.3	921	1.27
CB_URM(54a) ^2^	– ^2^	– ^2^	– ^3^	780 ^2^	– ^3^
CB_CFRP_S(54b)	1.93	0.23	718.5	970	1.35
CB_CFRP_L(56)	5.08	0.42	– ^3^	831	– ^3^
CB_URM(57)	2.76	1.16	313.0	480	1.54
CB_CFRP_W(58)	4.26	0.62	803.5	1230	1.53
CB_CFRP_S_R20(61)	10.76	1.07	488.3	720	1.48
CB_CFRP_S_R35(62)	5.97	0.68	497.6	870	1.75
CB_CFRP_S_R50(63)	8.21	0.34	509.2	950	1.87
CB_CFRP_W_R50(64)	11.46	3.06	680.6	1200	1.76
CB_CFRP_S_R85(65)	7.76	0.56	489.7	820	1.67
CB_CFRP_L_J3(66)	3.46	0.42	– ^3^	650	– ^3^
CB_CFRP_L_J2(67)	3.77	0.11	– ^3^	660	– ^3^
CB_CFRCM_W(68)	4.09	1.02	– ^3^	680	– ^3^
CB_URM(69)	3.18	0.1	269.9	660	2.45
CB_CFRP_S_H75_1/3(70)	4.39	0.43	367.5	839	2.29
CB_CFRP_S_H150_1/2(71)	4.91	0.05	378.4	720	1.90
CB_CFRP_S_H75_1/2(72)	6.31	1.16	301.2	690	2.29
CB_CFRCM_S(73)	5.52	0.83	– ^3^	678	– ^3^
CB_CFRP_S_R65(A02)	4.82	0.85	528.2	1061	2.01
CB_CFRP_S_R85(A03)	5.60	0.15	522.9	950	1.82

^1^ Last measured values before removal of the measuring gauges (due to their possible destruction); ^2^ Columns loaded only up to the appearance of first cracks (40%–60% of ultimate load); ^3^ There are no known formulas for determining the theoretical load-bearing capacity; ^4^ Theoretical load-bearing capacity pursuant to ČSN EN 1996 1-1 [30], and CNR-DT 200 R1/2013 [32].

**Table 6:** Summary of experimental loading results—stone columns.

Label	Ultimate vertical displacement ^1^ [mm]	Ultimate horizontal displacement ^1^ [mm]	*N*_theor_^2^ [kN]	*N*_exp_ [kN]	*N*_exp_/*N*_theor_
ST_URM_IREG(K01)	3.56	1.83	505.2	600	1.19
ST_URM_REG(K02)	3.42	0.22	1122.8	2280	2.03
ST_CFRP_S_IREG(K03)	5.45	1.14	1073.7	1360	1.27
ST_CFRP_S_REG(K04)	3.59	0.39	1899.0	2641	1.39
ST_CFRP_S_IREG(K07a)	6.17	1.09	600.0	1111	1.85
ST_URM_MIX_IREG(K09)	2.83	1.36	532.7	832	1.56
ST_CFRP_L_IREG(K13)	5.01	1.64	237.7	900	3.78
ST_CFRP_L_MIX_IREG(K15)	5.38	0.78	524.5	900	1.72

^1^ Last measured values before removal of the measuring gauges (due to their possible destruction); ^2^ Theoretical load-bearing capacity pursuant to ČSN EN 1996 1-1 [30], and CNR-DT 200 R1/2013 [32].
